# Useful Institutional Furniture

**Published:** 1900-12-15

**Authors:** 


### USEFUL INSTITUTIONAL FURNITURE.

THE selection of thoroughly good and at the same time useful office furniture is a problem that most of us are at one time or other of our lives called upon to grapple with. Desks of the right size and capacity, cabinets, and bookshelves require no little thought and anxiety to choose. Thomas Turner (Leicester), Limited, whose showrooms are at 44 Holborn Viaduct, and 9 Newark Street, Leicester, has done much to relieve the perplexity of the intending purchaser by his excellent examples in this line. Turning to the catalogue we find a vast assortment of desks, ornamental as well as useful, in all shapes, sizes, and material. The roll-top desk is undoubtedly the best for ordinary business purposes, as it locks a portion, or the whole of the desk as preferred, automatically, when the cover is drawn down. The model which perhaps recommends itself most to our fancy is that described as the "New Style Derby Standard High Grade Desk," No. 322 in the list: for completeness of arrangement and accuracy of detail we have rarely seen it equalled at the price. The pigeon-hole case has swell fronts, allowing sufficient extra depth for filing large documents. Six hardwood filing boxes are furnished with each desk, and there are spaces at either side of the upper case with movable partitions 16½ inches in height for books. It is also provided with swing arms at the sides, which can be thrown open as required to admit the light when writing. A somewhat smaller desk is that numbered 316: it is admirably adapted for home use. It has all the convenience of a large roll-top desk, but takes up a minimum of room with a maximum of capacity. It is provided with a "Yale" lock on the centre drawer, and when the cover descends it locks the entire desk with this one exception. A small roll-top desk is supplied for rooms of limited size, in light oak, at the very reasonable price of £5 10*s*.

The "Werniche" elastic bookcase is another speciality to which it is impossible to give too much emphasis. By an ingenious system of "units" this bookshelf is rendered capable of infinite expansion or contraction as desired. It need never be either too small or too large. In effect it consists of a series of small compartments designed to interlock with one another, in vertical and horizontal arrangement. They can be arranged to take in books of varying depths and sizes, and are readily adjustable to the required size. In fact, it is small enough for 10 or large enough for 10,000 books. It possesses the further advantage of looking always complete. As the library grows so does the bookshelf, but never till it is wanted; hence the economy in space. A further advantage is that the bookcase can be made to assume any shape that lends itself most conveniently to the size of the room. On much the same principle are the elastic cabinets, which are capable of expansion and adjustment in the same way. We all know how soon existing cabinets become too small, and it is not always either profitable or convenient to part with them, but by the "Globe Wernicke" system this is obviated by the power to increase the dimensions at will. We are greatly impressed by the completeness and thoroughness of all the above articles, and can confidently recommend them to the serious consideration of intending purchasers.

